# When “looks” can be deceiving – Internuclear ophthalmoplegia after mild traumatic brain injury: Case report and literature review

**DOI:** 10.1016/j.ijscr.2019.08.032

**Published:** 2019-09-11

**Authors:** Shaikh Hai, Adel Elkbuli, Kyle Kinslow, Mark McKenney, Dessy Boneva

**Affiliations:** a Department of Surgery, Kendall Regional Medical Center, Miami, FL, United States; b University of South Florida, Tampa, FL, United States

**Keywords:** CT, computed tomography, MRI, magnetic resonance imaging, INO, internuclear ophthalmoplegia, WEMINO, wall-eyed monocular internuclear ophthalmoplegia, WEBINO, wall-eyed bilateral internuclear ophthalmoplegia, MS, multiple sclerosis, MLF, medial longitudinal fasciculus, PPRF, paramedian pontine reticular formation, DAI, diffuse axonal injury, CN, cranial nerve, CVA, cerebrovascular accident, Blunt trauma, WEMINO, INO, Exotropia

## Abstract

**Introduction:**

Walled-eyed monocular internuclear ophthalmoplegia (WEMINO) syndrome is a sub-variant of internuclear ophthalmoplegia (INO) and involves the same clinical findings with the addition of exotropia of the ipsilateral eye. Causes typically include multiple sclerosis (MS) and ischemia (hemorrhagic or embolic) but can be secondary to blunt trauma as seen in our presented case.

**Presentation of case:**

A 27-year-old man presented with new-onset visual changes, diplopia, and strabismus following a motor vehicle collision. Physical exam showed left ocular exotropia and slight hypertropia on forward gaze with deficiency of left convergence and disconjugate eye movements on horizontal gaze with right nystagmus on rightward gaze. Imaging showed hyperintensities in the right middle cerebellar peduncle and left temporal-occipital white matter likely consistent with diffuse axonal injury but otherwise nonspecific. The patient was treated conservatively with left eyepatch and exhibited improvement of exotropia and diplopia at 1 week follow up.

**Discussion:**

Common causes of WEMINO syndrome include MS and ischemia with no prior reports, to our knowledge, being secondary to the blunt trauma seen in our case. Patients with WEMINO present with the typical signs of failure of ipsilateral adductive movement during lateral along with ipsilateral exotropia. Management involves treating the underlying disorder, if possible, with conservative measures with traumatic origins.

**Conclusion:**

Until now, WEMINO syndrome secondary to trauma has not been previously documented. Our patient was effectively treated with conservative measures alone.

## Highlights

• We report a case of WEMINO caused by mild traumatic brain injury, presenting with typical left ocular manifestations.
• CT scan of brain was noncontributory, but MRI managed to delineate the site of injury in the MLF in the left temporo-occipital white matter.
• Although more common in demyelinating diseases, clinicians should be cognizant of this syndrome in mild TBI patients that have ocular exotropia.

## 1 Introduction

Wall-eyed monocular internuclear ophthalmoplegia, called WEMINO syndrome and its bilateral homologue WEBINO syndrome are both sub-variants of internuclear ophthalmoplegia (INO). INO presents clinically as a failure of monocular adduction during attempted lateral gaze with simultaneous lateral nystagmus of the contralateral abducting eye. When instructed to look to their left, those with a **right** INO are able to laterally abduct their left eye but fail to adduct the right eye towards the nose. Additionally, the left abducting eye will exhibit nystagmus. Those with WEMINO exhibit a similar failure of monocular abduction with contralateral gaze but also have exotropia of the affected eye with primary gaze. Therefore, a patient with **right** WEMINO would be unable to adduct the right eye with left lateral gaze as well as having rightward deviation when instructed to focus frontward. WEBINO is the bilateral sibling of WEMINO and exhibits the same pathology except with involvement of both eyes (bilateral exotropia with failure to adduct both eyes to the midline). The basis of all INO types involve damage to the Medial Longitudinal Fasciculus (MLF), a white matter tract that connects the paramedian pontine reticular formation (aka the “horizontal gaze center”) to the medial rectus nuclei of the contralateral eye. This tract serves to connect the lateral gaze initiation of the paramedian pontine reticular formation (PPRF) via cranial nerve (CN) VI of one eye, with medial adduction of the contralateral eye via CN III to allow for synchronous eye movement towards a horizontal direction. MLF lesions of any etiology result in dissociation of medial rectus activation with lateral rectus movement and present with the clinical findings described above.

Causes of WEMINO and WEBINO are the same as those of general INO and include stroke, multiple sclerosis, tumors, infection, hydrocephalus, metabolic disturbance, toxins, degenerative disease, inflammatory disease, and trauma. However, by far the most common include multiple sclerosis and cerebrovascular accident (CVA) or stroke [1]. Here, we report an index case of WEMINO in a 27-year-old male status-post mild head trauma secondary to a motor vehicle accident. We also perused the literature for diagnostic approach, treatments, and prognosis. This case has been reported in line with the SCARE criteria [2].

## 2 Case presentation

A 27-year old male with no significant past medical history presented to emergency department (ED) of our Level 1 Trauma Center with a 1-day history of new-onset visual changes, diplopia, and strabismus following a motor vehicle collision. He was an unrestrained front seat passenger of a vehicle traveling approximately 40 mph when it was involved in a side impact on his side. While he reported a head strike from the initial impact, he did not lose consciousness. He was able to ambulate after the incident though he did experience unsteady gait and stumbling. He also stated that while visual blurriness was not initially present after the accident, onset began several hours afterwards and was associated with his left eye being deviated outwards and unable to move medially. Additional complaints at presentation to the ED included visual blurriness, vertical diplopia, left forehead pain, mild photophobia, strabismus, and improved but still present unsteady gate. He denied any significant headache or any focal numbness or weakness of his face or limbs at intake. His only past medical history was significant only for a concussion from playing football in the remote past.

Physical exam revealed a GCS of 15 and was significant for bilateral pupil exam normal, but left ocular extropia and slight hypertropia on forward gaze with deficiency of left convergence and disconjugate eye movements on horizontal gaze with nystagmus. Right ocular movement was not disturbed in any direction, but horizontal nystagmus appeared on rightward gaze. No ptosis was present in either eye. Visual acuity was grossly normal; visual fields showed no defects bilaterally; and normal finger-to-nose test bilaterally. Laboratory values were all within normal limits.

A non-contrast computed tomography (CT) brain showed possible small right frontal hemorrhagic contusion and small subarachnoid hemorrhage. CT of the facial bones was negative for cranio-facial fractures. Subsequent magnetic resonance imaging (MRI) of the Brain (Fig. 2, Fig. 3) revealed T2/FLAIR hyperintensities in the right middle cerebellar peduncle and left temporal-occipital white matter. While these findings were nonspecific, they may be seen with demyelinating diseases, diffuse axonal injury (DAI), or chronic small vessel ischemic disease. Other less common etiologies would include various infectious or inflammatory processes and vasculitis.

Our traumatic brain injury neurology team was consulted and concluded that given the patient's presentation, post-traumatic DAI as the etiology. A follow up non-contrast CT brain was the next day showed resolution of the punctate right frontal lobe contusion and the SAH. They also ordered a CT Angiogram of brain to rule out a vascular etiology and to shed light on his symptomatology. The results showed no hemodynamically significant stenosis, no aneurysmal dilatation or dissection of the anterior and posterior cerebral circulation. The anterior and posterior communicating arteries were unremarkable, the arterial Circle of Willis complete and the brain vessels intact and patent.

The ophthalmology evaluation revealed normal visual acuity bilaterally, normal visual fields bilaterally, and normal intraocular pressure. Slit lamp and dilated pupillary examinations were unremarkable. After group discussions with neuro-radiology, ophthalmology, and trauma neurology the patient was diagnosed the patient with Wall-Eyed Monocular Internuclear Ophthalmoplegia (WEMINO), a rare subtype of Internuclear Ophthalmoplegia (INO). Our patient was treated conservatively, with short-term application of left eye patch and discharged home. On follow-up visit in the trauma clinic 1 week later, it was found that his left ocular exotropia was less pronounced, and he stated that his diplopia was improving as well. After 12 week follow up, the patient had complete recovery of eye function with correction of the exotropia and was back to work without issues.

## 3 Discussion

### 3.1 Pathogenesis and associated conditions

**Fig. 1:**
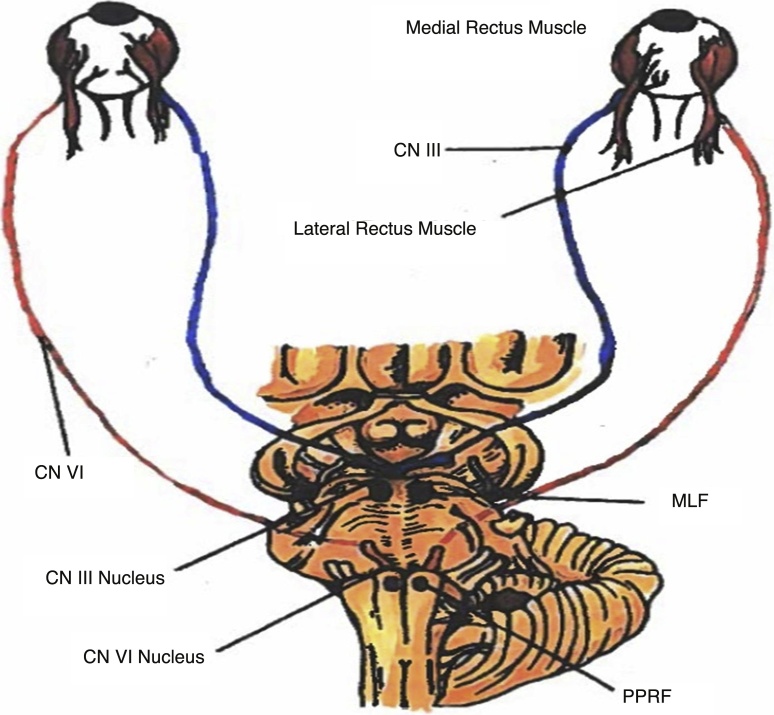
Showing the Cranial nerves and various pathways involved in the control of eye movements. CN (Cranial Nerve), MLF (Medial Longitudinal Fasciculus), PPRF (Paramedian Pontine Reticular Formation).

INO is an ocular movement disorder classified as a pre-nuclear cerebral disorder and known to be associated with damage to the medial longitudinal fasciculus (MLF). The MLF pathway carries inter-nuclear neurons to connect nuclei of the brain stem, including the nucleus of cranial nerve (CN) VI, also known as abducens nerve, in the pons to the contralateral sub-nucleus of CN III, also known as the oculomotor nerve, in the midbrain that innervates the medial rectus muscle. (See Fig. 1). Ultimately, this pathway functions to allow synchronous eye movements with intentional and reflex horizontal gaze [3].

**Fig. 2:**
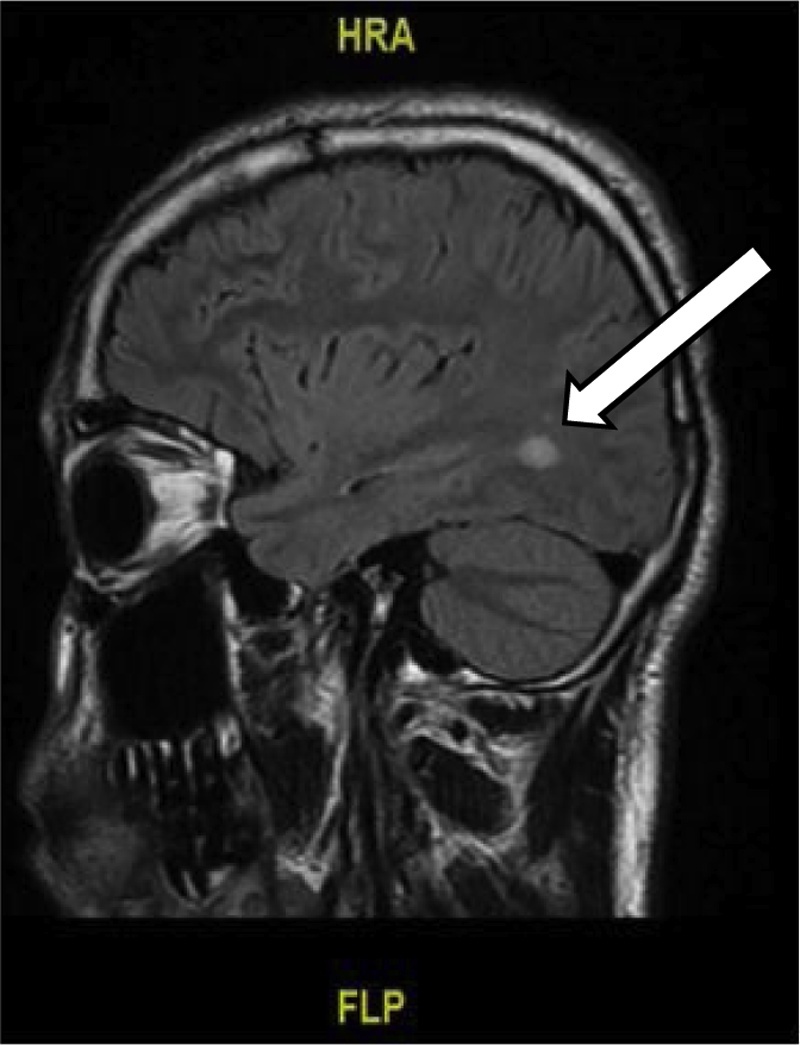
Sagittal view MRI brain showing T2/FLAIR hyperdensity in the left temporal-occipital white matter. This non-specific finding can be seen with demyelinating disease or rarely trauma.

**Fig. 3:**
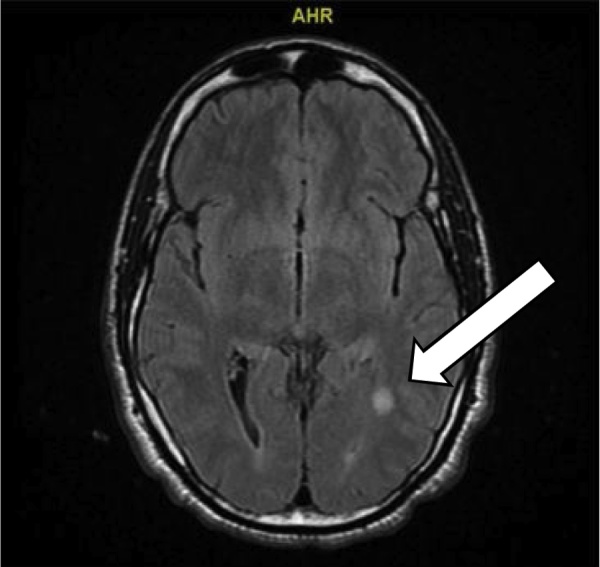
Coronal view showing the same "spot" in the left temporal- occipital white matter. This is a 10 × 8 mm T2/FLAIR white matter hyperintensity at the junction of the left temporal and occipital lobes. The lesion is isointense, does not enhance, did not restrict and does not demonstrate blooming artifact (to suggest hemorrhage).

Historically, specialists have separated INO into anterior (front) and posterior (back) varieties based on location of the lesion and clinical sequelae, but MRI scans have shown that this classification is unreliable. It is classically characterized by presence of impaired adduction of the ipsilateral eye with nystagmus of the contralateral abducting eye. The pathogenesis leading to the extropia manifestations of WEMINO syndrome is unclear with multiple potential origins suggested in previous studies. According to a literature review conducted by Wu et al., either direct oculomotor nucleus or pontine lesions can explain the exotropia during primary gaze. The former being responsible for medial rectus instability with the latter potentially leading to “overstimulation” of the ipsilateral abducens as proposed by Komiyama et al. [1,4]. However, reports have shown WEBINO in patients with intact ocular motor nuclei which suggests that multiple origins may uniquely contribute to the similar clinical findings seen, overall. From an ischemic origin, medial dorsal pons infarction has also been reported as a proposed etiology for exotropia symptoms according to Sakamoto et al. [5]. Some histologic studies have shown that small groups of medial rectus neurons were found in MLFs implying that damage to the MLF (causing the INO) may also have concomitant damage to medial rectus activation resulting in exotropia [1]. Of note, there are two other syndromes supposedly involving a combination of injury to the medial longitudinal fasciculus and exotropia, and should be included in the differential diagnosis; these are paralytic pontine exotropia (PPE) and non-paralytic pontine exotropia (NPPE). However, WEMINO syndrome can be discriminated from both PPE and NPPE, because the latter show exotropia on the side contralateral to the injured medial longitudinal fasciculus while WEMINO syndrome exhibits ipsilateral exotropia.

### 3.2 Etiologies

The most frequent causes of WEMINO and WEBINO include cerebrovascular accident (CVA)/ stroke and demyelinating disorders such as Multiple Sclerosis (MS), with infection, tumors, vasculitis, and iatrogenic injury from neurosurgical procedures being less common [6]. As WEMINO is usually associated with CVA, to date, our patient is the first described case of WEMINO developing following minor blunt trauma to the head. While Keane et al. reported 16 of 410 studied cases of INO being secondary to blunt trauma, the additional findings of exotropia required for WEMINO diagnosis are not well documented in a similar context [7]. Furthermore, neither cases of WEMINO nor WEBINO have been reported in literature to develop following any trauma, and are almost always attributable to a cerebrovascular accident, whether thrombotic, embolic, or hemorrhagic [8]. Diagnosis is usually clinical, with MRI used to determine any potential causative lesions. In our patient, the clinical findings observed were not concordant with what was seen on imaging, as MRI was indicative of small focal regions of DAI that did not involve the MLF or other ocular movement tracts. This aligns with current literature which suggest that additional histologic investigations may be necessary to further ascertain the specific pathogenesis pertaining to this condition [1,9].

### 3.3 Treatment

Treatment of WEMINO/WEBINO should be dictated by the discovered/presumed underlying etiology. In patients with MS, high-dose intravenous steroids may help alleviate symptoms by accelerating the resolution of the causative plaque. Dalfampridine or teriflunomide may be utilized as disease modifying therapy to improve axonal function in MS in the case that interferon and Glatiramer are not available. Infectious causes should be dealt with targeted pharmacotherapy and metabolic or toxic causes managed accordingly. INO due to trauma or demyelination usually resolves spontaneously over 3–6 months while INO secondary to cerebral infarction or tumor however is typically permanent. As shown in our presented case, treatment of WEMINO and WEBINO due to trauma is typically conservative, and aimed at symptom control using Fresnel prisms lenses or ipsilateral eyepatch as well as treatment of the underlying cause, if possible. Those patients suffering from intolerable diplopia and persistent visual derangement may benefit from botulinum toxin injections of opposing extraocular muscles (i.e. Ipsilateral lateral rectus) to minimize exotropia symptoms. Surgical correction is typically last-line therapy to utilize in persisting diplopia due to permanent causes, as well as in patients that suffer from persistent deficits [1,6].

## 4 Conclusion

To our knowledge, we report the first case of WEMINO syndrome secondary to a blunt trauma origin. Our patient initially presented with left ocular exotropia and slight hypertropia on forward gaze with deficiency of left convergence and disconjugate eye movements on horizontal gaze with right ocular nystagmus on rightward gaze. MRI imagining was correlative with diffuse axonal injury. Our patient was treated conservatively with a left eye patch and showed complete clinical resolution at 12-week follow up.

## Funding

None.

## Ethical approval

This report was conducted in compliance with ethical standards. Informed written consent has been obtained and all identifying information is omitted.

## Consent

Informed written consent has been obtained and all identifying information is omitted.

## Author’s contribution

Shaikh Hai, Adel Elkbuli, Dessy Boneva, Kyle Kinslow, Mark McKenney– Conception of study, acquisition of data, analysis and interpretation of data.

Shaikh Hai, Adel Elkbuli, Dessy Boneva, Kyle Kinslow - Drafting the article.

Shaikh Hai, Dessy Boneva– Management of case.

Shaikh Hai, Adel Elkbuli, Kyle Kinslow, Dessy Boneva, Mark McKenney – Critical revision of article and final approval of the version to be submitted.

## Registration of research studies

This is a case report study.

## Guarantor

Dessy Boneva.

Mark McKenney.

Shaikh Hai.

## Provenance and peer review

Not commissioned, externally peer-reviewed.

## Declaration of Competing Interest

None.
